# Huanshaodan regulates microglial glucose metabolism reprogramming to alleviate neuroinflammation in AD mice through mTOR/HIF-1α signaling pathway

**DOI:** 10.3389/fphar.2024.1434568

**Published:** 2024-07-26

**Authors:** Congcong Shang, Yunfang Su, Jinlian Ma, Zhonghua Li, Pan Wang, Huifen Ma, Junying Song, Zhenqiang Zhang

**Affiliations:** ^1^ Collaborative Innovation Center of Research and Development on the Whole Industry Chain of Yu-Yao, Henan University of Chinese Medicine, Zhengzhou, Henan, China; ^2^ The First Affiliated Hospital of Henan University of Chinese Medicine, Zhengzhou, China

**Keywords:** Huanshaodan, Alzheimer’s disease, neuroinflammation, mTOR/HIF-1α signaling pathway, microglial glucose metabolism reprogramming

## Abstract

Abnormal glucose metabolism in microglial is closely associated with Alzheimer’s disease (AD). Reprogramming of microglial glucose metabolism is centered on regulating the way in which microglial metabolize glucose to alter microglial function. Therefore, reprogramming microglial glucose metabolism is considered as a therapeutic strategy for AD. Huanshaodan (HSD) is a Chinese herbal compound which shows significant efficacy in treating AD, however, the precise mechanism by which HSD treats AD remains unclear. This study is aim to investigate whether HSD exerts anti-AD effects by regulating the metabolic reprogramming of microglial through the mTOR/HIF-1α signaling pathway. SAMP8 mice and BV2 cells were used to explore the alleviative effect of HSD on AD and the molecular mechanism *in vivo* and *in vitro*. The pharmacodynamic effects of HSD was evaluated by behavioral tests. The pathological deposition of Aβ in brain of mice was detected by immunohistochemistry. ELISA method was used to measure the activity of HK2 and the expression of PKM2, IL-6 and TNF-α in hippocampus and cortex tissues of mice. Meanwhile, proteins levels of p-mTOR, mTOR, HIF-1α, CD86, Arg1 and IL-1β were detected by Western-blot. LPS-induced BV2 cells were treated with HSD-containing serum. The analysis of the expression profiles of the CD86 and CD206 markers by flow cytometry allows us to distinguish the BV2 polarization. Glucose, lactic acid, ATP, IL-6 and TNF-α levels, as well as lactate dehydrogenase and pyruvate dehydrogenase activities were evaluated in the BV2. Western-blot analysis was employed to detect mTOR, p-mTOR, HIF-1α and IL-1β levels in BV2. And the mTOR agonist MHY1485 (MHY) was chosen to reverse validate. In this study, it is found that HSD improved cognitive impairment in SAMP8 mice and reduced Aβ deposition, suppressed the levels of glycolysis and neuroinflammation in mice. In LPS-induced BV2 cells, HSD also regulated glycolysis and neuroinflammation, and suppressed the mTOR/HIF-1α signaling pathway. More importantly, these effects were reversed by MHY. It is demonstrated that HSD regulated microglial glucose metabolism reprogramming by inhibiting the mTOR/HIF-1α signaling pathway, alleviated neuroinflammation, and exerted anti-AD effects. This study provided scientific evidence for the clinical application of HSD for treating AD.

## 1 Introduction

According to the World Health Organization, as the global population ages, the incidence of Alzheimer’s disease (AD) is increasing year by year (Fan et al., 2022). AD typically manifests as a decline in cognitive abilities, and even in later stages of the disease, there may be a decrease in motor function and other abnormal mental behaviors (Lane et al., 2018). The primary pathological characteristics of AD are the deposition of β-amyloid (Aβ) protein, forming extracellular senile plaques, and the hyperphosphorylation of tau protein, leading to neurofibrillary tangles and neuronal loss (Gong et al., 2018). The dissection of the brain in AD patients revealed parenchymal atrophy, in addition to the main pathological features of AD, activated microglial could also be observed under the microscope (DeTure and Dickson, 2019). Evidently, microglia is intimately linked to the occurrence and development of AD (Bohlen et al., 2019).

Microglia exists in the brain parenchyma and account for 10%–15% of the total number of glial cells (Nayak et al., 2014) which play important roles in maintaining the health of the brain (Bilimoria and Stevens, 2015). Quiescent microglia can secrete various neurotrophic factors to participate in the development of neurons, such as basic fibroblast growth factor (bFGF) and nerve growth factor (NGF) (Araujo and Cotman, 1992; Frade López and Barde, 1998; Ueno et al., 2013). During the evolution of AD development, the immunometabolism of microglia is a significant component of the inflammatory reaction (Shippy and Ulland, 2020). The metabolic reprogramming of microglial plays a crucial role in regulating their functions (Bernier et al., 2020). During the metabolic reprogramming of microglia, M1-type microglia produce the Warburg effect, in which cellular metabolism shifts from oxidative phosphorylation (OXPHOS) to aerobic glycolysis which results in an accelerated rate of glucose metabolism, thus facilitates microglial migration, proliferation, and phagocytosis, among other functions (Baik et al., 2019). The activation of mammalian target of rapamycin (mTOR)/hypoxia inducible factor-1α (HIF-1α), leading to metabolic reprogramming, is an important mechanism for microglial M1 phenotype polarization (Baik et al., 2019). mTOR positively enhances HIF-1α translation, which leading to glycolytic metabolism and promoting AD progression (Soto-Heredero et al., 2020; March-Diaz et al., 2021). Furthermore, the accumulation of HIF-1α is recognized to trigger the generation of the pro-inflammatory cytokine IL-1β (York et al., 2021). The M2 phenotype is another classic activation pathway of microglial, which can promote tissue repair and protect the body from inflammatory damage (Cherry et al., 2014). M1/M2 paradigm is used to explain extreme inflammatory reactions, elucidating the activated state of microglial in the central nervous system (Tang and Le, 2016). Research has demonstrated that blocking the mTOR/HIF-1α signaling pathway can significantly suppress neuroinflammation by regulating glucose metabolism reprogramming (Zhao et al., 2021). Therefore, modulation of mTOR/HIF-1α-mediated reprogramming of microglial cell glucose metabolism to attenuate neuroinflammation is a potential mechanism for the treatment of AD.

It has a long history for traditional Chinese medicine (TCM) in the recognition of AD. Chinese herbal medicine, being purely natural, entails minimal side effects, with extensive clinical experience in treating AD over the long term. AD belongs to “Chidai” in TCM and its core pathogenesis is attributed to “kidney deficiency and marrow depletion”. Kidney tonification is the most basic principle in treating AD (Tian and Shi, 2018). Huanshaodan (HSD) originated from the Song Dynasty’s “Hongshi Jiyuan Fang” which is a formula for tonifying the kidney and invigorating the spleen. It consists of Chinese yam, Achyranthes, Poria, Eucommia bark, Goji berry, Morinda root, Rehmannia, Acorus, Polygala, Fennel, Chushizi, Cistanche, Dogwood, Schisandra fruit and Jujube. Clinical studies indicate that HSD has anti-aging and memory-enhancing functions (Yang et al., 2020), however, the underlying mechanism of HSD in the treatment of AD needs to be clarified. Studies have shown that single herbal medicine Acorus in HSD exerts anti-AD effects by alleviating neuroinflammation (Balakrishnan et al., 2022; Xu et al., 2023), Cistanche can regulate neuroinflammation mediated by microglia (Liao et al., 2023). Poria can reduce the production of Aβ and relieve the pathology of AD (Sun et al., 2021), Goji berry alters macrophage glycolysis, inhibits M1-type differentiation and reduces HIF-1α formation to combat lipopolysaccharide (LPS)-induced neuroinflammation (Ding et al., 2021). However, it remains unclear whether HSD inhibits neuroinflammation and exerts anti-AD effects by affecting the reprogramming of microglial glucose metabolism through the mTOR/HIF-1α pathway. Therefore, in this study, SAMP8 mice and BV2 cells were used to investigate the potential mechanism of HSD in the treatment of AD.

## 2 Materials and methods

### 2.1 Materials

HSD (State Food and Drug Administration (SFDA) approval number: Z20025017) was purchased from China Chongqing Taiji Group TongJunGe Pharmaceutical Co., LTD. Donepezil hydrochloride (SFDA approval number: H20010723) was sourced from Eisai China. The main materials that were used in this study were LPS (L8880, Solarbio), MHY1485 (MHY) (M9050-5MG, AbMole), RIPA buffer (high) (R0010, Solarbio), primary antibodies β-Actin and CD86 (42kDa, BM0627; 70kDa, BM4121, BOSTER), Arg1 and mTOR (40kDa, GB11285-100; 289kDa, GB111839-100, Servicebio), p-mTOR and HIF-1α (289kDa, 5536T; 120kDa, 48085S, Cell Signaling Technology), IL-1β (31kDa, 16806-1-AP, Proteintech), Horseradish peroxidase (HRP)-linked secondary antibodies against rabbit and mouse IgG of the primary antibodies (ZB-2301; ZB-2305, Beijing Zhong Shan-Golden Bridge Biological Technology), Glucose (GLU) Assay Kit, Lactic Acid (LD) assay kit, ATP assay kit, Lactate dehydrogenase (LDH) assay kit (A154-1-1; A019-2-1; A095-1-1; A020-2-2, Nanjing Jiancheng Bioengineering Institute), Pyruvate Dehydrogenase (PDH) Activity Assay Kit (E-BC-K650-M, Elabscience), BCA Protein Assay Kit (PC0020, Solarbio), ELISA Kit for Hexokinase 2 (HK2) activity based on enzyme standard of HK2, ELISA Kit for Tumor Necrosis Factor alpha (TNF-α) (MM-45418M1; MM-0132M1, MEIMIAN), ELISA Kit for Interleukin 6 (IL-6) (E-MSEL-M0001, Elabscience) and ELISA Kit for Pyruvate kinase isozymes M2 (PKM2) (SEA588Mu, Cloud Clone Corp), Cell Staining Buffer, PE Anti-Mouse F4/80 Antibody [CI:A3-1], PE/Cyanine7 Anti-Mouse CD86 Antibody [GL-1], Intracellular Fixation/Permeabilization Buffer Kit and APC Anti-Mouse CD206 Antibody (E-CK-A107; E-AB-F0995D; E-AB-F0994H; E-CK-A109; E-AB-F1135E, Elabscience).

### 2.2 Animals and drug treatment

Six-month-old male SAMP8 and SAMR1 mice were purchased from Beijing Vital River Laboratory Animal Technology Co., LTD. [license number was SCXK (jing) 2016-0010]. The mice were randomly divided into 4 groups: control group, model group, Donepezil group (0.747 mg/kg/d) and HSD group [2.7 g/kg/d, based on our previous study (Su et al., 2023)] with 12 mice in each group. HSD and donepezil were dissolved in 0.9% saline and administered by continuous gavage for 2 months. A same volume of 0.9% saline was given to the mice in control and model groups. The experimental procedures were carried out in accordance with the principles of experimental animal welfare (Ethical approval number of experimental animals: DWLLGZR202202147).

### 2.3 Behavior experiments

Morris water maze, open-field and Y-maze experiments were conducted 5 days before the end of the animal experiments. Morris water maze test: In 5 consecutive days, mice were gently placed in water to record the time it took for them to climb onto a platform (i.e., escape latency). After removing the platform, mice were placed into the water from the same point. The time spent in the target quadrant and the number of platform crossings were recorded during 60 s of free exploration. Open-field experiment: The mice were placed in the center of the square and allowed to move freely for a period of five minutes. The system records the total distance traveled by mice, the duration of their sojourn in the central zone, and the frequency of grooming instances. Y-maze experiment: Place the mice one by one at the junction of the Y-maze, allow them to freely explore for 5 min, and record the total number of entries into the arms and the spontaneous alternation rate (Zheng et al., 2022). The mice were euthanized at the end of the behavioral study and the brain tissue was utilized for subsequent experiments.

### 2.4 IHC staining

The whole mouse brain was fixed in 4% paraformaldehyde, embedded in paraffin after sectioning, and Aβ_1-42_ antibody was used as primary antibody for routine immunohistochemical staining (Hussaini et al., 2022). The images were captured under a microscope (Pannoramic MIDI, 3DHISTECH), and the average optical density value of Aβ_1-42_ was calculated by ImageJ software.

### 2.5 Enzyme-linked immunosorbent assay (ELISA)

To further study the activity of HK2 and the expression of PKM2, IL-6 and TNF-α in hippocampus and cortex of mice, and the expression of IL-6 and TNF-α in BV2 cells. The BV2 cells, hippocampus and cortex tissues were ground and centrifuged, then supernatants were collected, and a BCA Protein Assay Kit was used to measure the protein concentration of the supernatant. The activity of HK2 and the expression of PKM2 in the supernatants were detected using ELISA Kits. Finally, the corresponding protein concentrations were compared and analyzed.

### 2.6 HSD-containing serum preparation and intervention in LPS-induced BV2 cells

The HSD-containing serum was prepared using male KM mice of (40 ± 5) g weight which were obtained from Beijing Vital River Laboratory Animal Technology Co., LTD. [license number was SCXK (zhe) 2020-0002]. The animals were housed in the Animal Experiment Center and the experiment was started after 1 week of acclimatization. HSD was dissolved in sterile 0.9% saline at a dose of 5.4 g/kg/d and administered to the mice by gavage for 7 days. Three hours after the last dose of medication, blood samples were collected from the eyeballs (fasted for 12 h before blood collection), standard serum collection and inactivation, filtered through a 0.22 μm filter, dispensed and stored in a −80°C refrigerator.

The BV2 cells (ATCC, US) were used for *in vitro* experiments, which were divided into 4 groups, the control group, the model group (LPS, 0.5 mg/mL), the HSDcontaining serum-treated group (14% HSD-containing serum, based on our preliminary study), and the reverse validation group (14% HSD-containing serum +8 μM MHY). The BV2 cells in logarithmic phase were induced by LPS in 6-well plate, and the HSD-containing serum was introduced without or with mTOR agonist MHY to continue treatment of the cells for 24 h. The cells were harvested and used for subsequent experiments.

### 2.7 Cell labeling for flow cytometry, cell GLU, LD, ATP, LDH, and PDH testing

BV2 and M1-type BV2 were respectively labeled with PE Anti-Mouse F4/80 and PE/Cyanine7 Anti-Mouse CD86, respectively, using Cell Staining Buffer as cell buffer and incubated for 30 min at 4°C, protected from light. The Cells were fixed with Fixation Buffer for 40 min at room temperature, protected from light. Subsequently, the cells were incubated with Permeabilization Working Solution and APC Anti-Mouse CD206 Antibody labeled with M2-type BV2 at room temperature and protected from light for 30 min, and then centrifuged to collect the cells. After resuspension, the cells were quantitatively analyzed and sorted by flow cytometry (BD FACSCanto™ II, BD Biosciences), and finally, the ratio of CD86 to CD206 was calculated for each group and normalized to assess the degree of polarization of BV2 (Zhu et al., 2020).

*In vitro* BV2 cells were collected after ultrasonic crushing and centrifugation to take the supernatant. According to the instructions of the method using GLU test Kit, LD test kit, ATP content test kit, LDH assay kit and PDH colorimetric test Kit to detect the expression levels of GLU, LD, and ATP, as well as the activity of LDH and PDH enzyme in the supernatant. The OD values were measured using a spectrophotometer, and the numerical values were calculated and compared with the corresponding protein concentrations measured by the BCA Protein Assay Kit.

### 2.8 Western blot

Mice hippocampus and cortex tissues were collected and added with RIPA high efficiency lysate buffer, grinded in a grinder at 4°C, stayed on ice for 30 min, centrifuged at 12,000 rpm for 15 min at 4°C, and the proteins were collected, and the protein concentration was detected by BCA kit. 20 μg of proteins per sample were separated on 10% sodium dodecyl sulfate polyacrylamide gel, and subsequently transferred to PVDF membrane, which were sealed with skim milk powder for 2 h and incubated overnight at 4°C with primary antibodies against CD86, Arg1, IL-1β, mTOR, p-mTOR, and HIF-1α. The membranes were washed three times and then incubated with horseradish enzyme-labeled goat anti-mouse or rabbit IgG secondary antibody at room temperature for 1 h. After washing the membrane three times, the bands were photographed (ChemiDoc™ MP Imaging System, Bio Rad) and quantified using ImageJ analysis software, and β-Actin was used as an internal control for data analysis (Mandler et al., 2014).

Cells *in vitro* experiment was collected and subjected to Western-blot for IL-1β, mTOR, p-mTOR, and HIF-1α as described above, and finally β-Actin was used as an internal control for data analysis.

### 2.9 Statistical analysis

GraphPad Prism software was used for statistical analysis. Two-factor ANOVA was used to analyze escape latency for behavioral experiments, and one-factor ANOVA was used to analyze protein blotting, ELISA, flow cytometry, GLU, LD, ATP, LDH, and PDH data. Data are expressed as mean ± standard error of mean (mean ± SEM).

## 3 Results

### 3.1 HSD improves cognition and learning memory in SAMP8 mice

**FIGURE 1:**
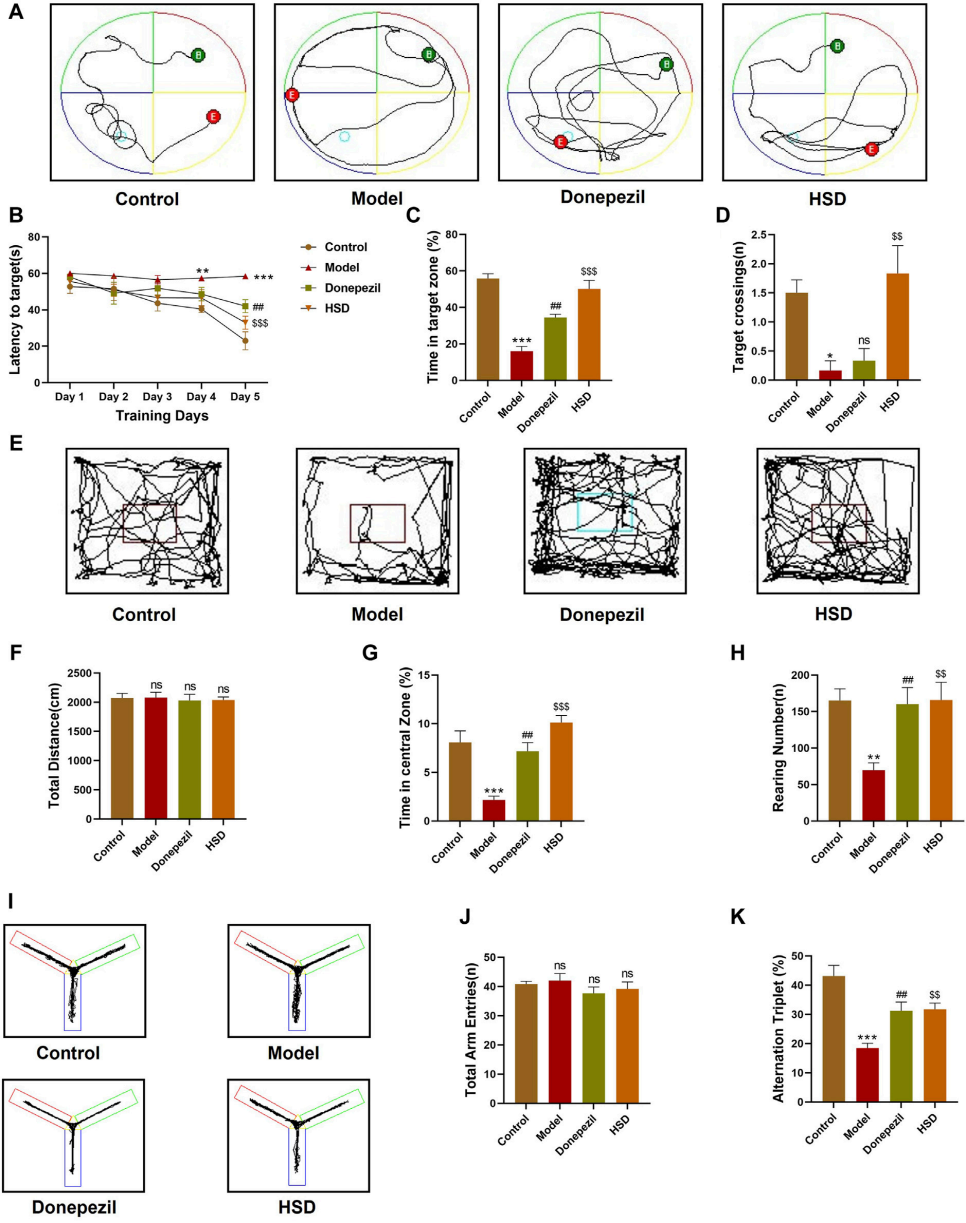
Behavioral experiment. **(A)** Morris water maze swimming trajectories of mice in each group; **(B)** Morris water maze evasion latency; **(C)** Percentage of time in target quadrant in the Morris water maze; **(D)** The number of platform crossings in Morris water maze; **(E)** Open-field activity trajectories of mice in each group; **(F)** Total moving distance of mice in open-field test; **(G)** Percentage of time spent in the central area of mice in open-field test; **(H)** Grooming times of mice in open-field test; **(I)** Y maze activity trajectory of mice in each group; **(J)** The total number of Y maze arm entries of mice in Y maze test; **(K)** Spontaneous alternation accuracy of mice in Y maze. Model group compared with the control group ^*^ *p* < 0.05, ^**^ *p* < 0.01, ^***^ *p* < 0.001, Donepezil group compared with the model group ^#^ *p* < 0.05, ^##^ *p* < 0.01, ^###^ *p* < 0.001, HSD group compared with the model group ^$^ *p* < 0.05, ^$$^ *p* < 0.01, ^$$$^ *p* < 0.001.

In order to investigate whether HSD improves the learning and spatial exploration abilities of SAMP8 mice, Morris water maze experiment was conducted. The trajectories of activity in the Morris water maze of the mice in each group are shown in Figure 1A. The results demonstrated that, the escape latency of the model group mice was significantly longer from the fourth day compared to the control group (*p* < 0.01). The escape latency on the fifth day was found to be significantly shorter in the Donepezil group and the HSD groups compared with the model group (*p* < 0.01, *p* < 0.001) (Figure 1B). On the final day of the platform exploration experiment, the proportion of time spent in the target quadrant and the number of platform crossings were significantly reduced in the model group than that in the control group (*p* < 0.001, *p* < 0.05), the proportion of time spent in the target quadrant significantly increased in the Donepezil group and the HSD groups compared with the model group (*p* < 0.01, *p* < 0.001). The comparison of platform crossing number between the Donepezil group and the model group was no statistically significance (*p* > 0.05), and the amount of platform crossing was significantly increased in the HSD group compared to the model group (*p* < 0.01) (Figures 1C, D). Mice open-field activity tracks for each group were shown in Figure 1E. The open-field experiment showed that there was no significant difference between the total distance traveled by the groups (*p* > 0.05) (Figure 1F). Compared with the control group, the percentage of time spent in the central area and the number of grooming were significantly reduced in the model group (*p* < 0.001, *p* < 0.01). While the percentage of time spent in the central area and the number of grooming were significantly increased in the Donepezil group and the HSD group compared to the model group (*p* < 0.01, *p* < 0.001) (Figure 1G). The number of grooming sessions was significantly increased in the Donepezil group and the HSD group compared to the model group (*p* < 0.01, *p* < 0.01) (Figure 1H). Furthermore, Figure 1I was Y-maze activity course of mice in each group. There was no difference in the total number of arm entries in each group in the Y-maze test (*p* > 0.05) (Figure 1J). Compared with the control group, the spontaneous alternation rate was significantly lower in the model group mice (*p* < 0.001), which was significantly increased in the donepezil and HSD groups compared to the model group (*p* < 0.01, *p* < 0.01) (Figure 1K).

### 3.2 HSD reduces Aβ deposition in the brain of SAMP8 mice

**FIGURE 2:**
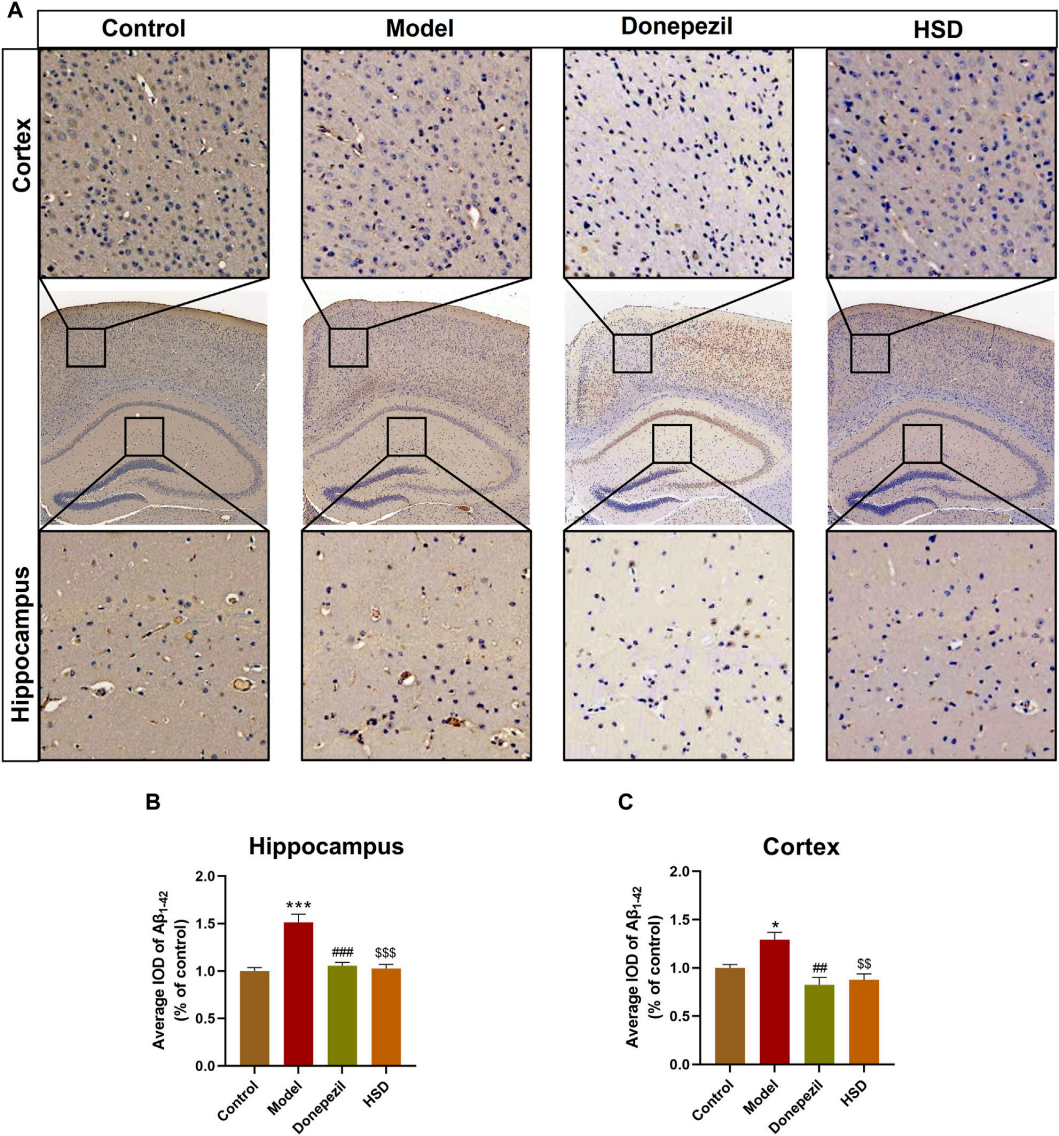
IHC staining of Aβ_1-42_ in hippocampus and cortex of SAMP8 mice. **(A)** IHC images of Aβ_1-42_ in hippocampus and cortex of SAMP8 mice (Magnification ×50, partial enlarged is Magnification ×300); **(B)** Aβ deposition level in hippocampus of mice in each group; **(C)** Aβ deposition level in cortex of mice in each group. Model group compared with the control group ^*^ *p* < 0.05, ^**^ *p* < 0.01, ^***^ *p* < 0.001, Donepezil group compared with the model group ^#^ *p* < 0.05, ^##^ *p* < 0.01, ^###^ *p* < 0.001, HSD group compared with the model group ^$^ *p* < 0.05, ^$$^ *p* < 0.01, ^$$$^ *p* < 0.001.

Aβ plaques are an important neuropathological marker in AD. Immunohistochemical results showed that Aβ plaques in the hippocampus and cortex of the model group were markedly increased in the control group (*p* < 0.001, *p* < 0.05), Aβ plaques in the hippocampus and cortex were substantially lower in the Donepezil group and the HSD group compared to the model group (*p* < 0.001, *p* < 0.001, *p* < 0.01, *p* < 0.01) (Figures 2A–C).

### 3.3 HSD reduces neuroinflammation in SAMP8 mice

**FIGURE 3:**
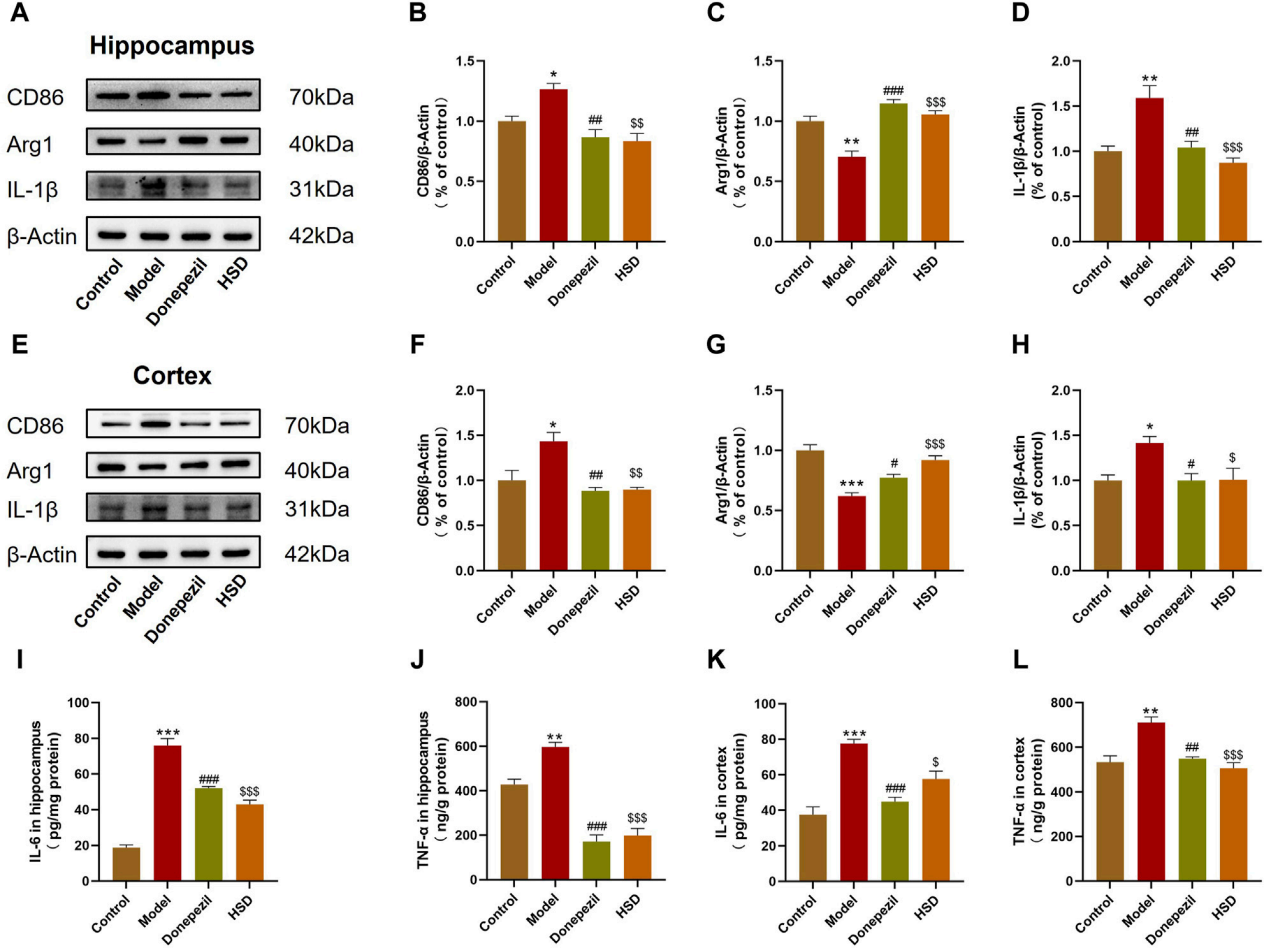
Expression levels of CD86, Arg1 and IL-1β protein in hippocampus and cortex of SAMP8 mice detected by Western-blot, the expression levels of IL-6 and TNF-α in hippocampus and cortex of SAMP8 mice by ELISA. **(A,E)** Blotting of CD86, Arg1 and IL-1β protein in hippocampus and cortex of mice in each group; **(B–D)** Expression of CD86, Arg1 and IL-1β protein in hippocampus of mice in each group; **(F–H)** Expression of CD86, Arg1 and IL-1β protein in cortex of mice in each group; **(I–L)** Expression of IL-6 and TNF-α protein in hippocampus and cortex of mice in each group. Model group compared with the control group ^*^ *p* < 0.05, ^**^ *p* < 0.01, ^***^ *p* < 0.001, Donepezil group compared with the model group ^#^ *p* < 0.05, ^##^ *p* < 0.01, ^###^ *p* < 0.001, HSD group compared with the model group ^$^ *p* < 0.05, ^$$^ *p* < 0.01, ^$$$^ *p* < 0.001.

The polarization of microglia and neuroinflammation-related proteins in the brain tissue were further detected by Western blot bands are shown in Figures 3A, E. The data indicated that the expression of CD86 was significantly elevated in the hippocampus and cortex of model group compared with the control group (*p* < 0.05, *p* < 0.05), with the expression of Arg1 was significantly reduced (*p* < 0.01, *p* < 0.001). Comparing with the model group, the expression of CD86 in the hippocampus and cortex was significantly reduced both in the Donepezil group and the HSD group (*p* < 0.01, *p* < 0.01, *p* < 0.01, *p* < 0.01) (Figures 3B, F), while the expression of Arg1 was significantly increased (*p* < 0.001, *p* < 0.05, *p* < 0.001, *p* < 0.001) (Figures 3C, G). Expression level of IL-1β was significantly higher in the hippocampus and cortex of the model group compared with the control group (*p* < 0.01, *p* < 0.05) which was significantly reduced both in the Donepezil group and the HSD group compared with the model group (*p* < 0.01, *p* < 0.05, *p* < 0.001, *p* < 0.05) (Figures 3D, H). Expression level of IL-6 was significantly higher in the hippocampus and cortex in the model group compared with the control group (*p* < 0.001, *p* < 0.001), while which was significantly reduced both in the Donepezil group and the HSD group compared with the model group (*p* < 0.001, *p* < 0.001, *p* < 0.001, *p* < 0.05) (Figures 3I, K). Similarly, the expression level of TNF-α was significantly higher in the hippocampus and cortex in the model group compared with the control group (*p* < 0.01, *p* < 0.01) which was significantly reduced both in the Donepezil group and the HSD group compared with the model group (*p* < 0.001, *p* < 0.01, *p* < 0.001, *p* < 0.001) (Figures 3J, L).

### 3.4 HSD inhibits the activity of glycolysis-related enzyme HK2 and the expression level of PKM2 in the brain of SAMP8 mice

**FIGURE 4:**
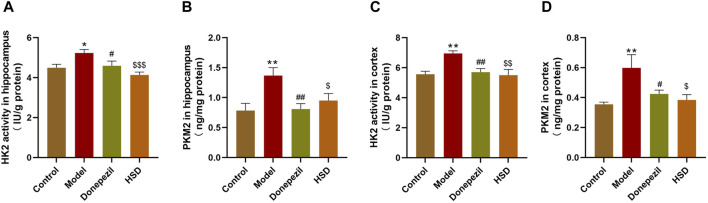
The activity of HK2 and the expression level of PKM2 in hippocampus and cortex of SAMP8 mice. **(A,B)** The activity of HK2 and the expression of PKM2 in the hippocampus of mice in each group; **(C,D)** The activity of HK2 and the expression of PKM2 in the cortex of mice in each group. Model group compared with the control group ^*^ *p* < 0.05, ^**^ *p* < 0.01, ^***^ *p* < 0.001, Donepezil group compared with the model group ^#^ *p* < 0.05, ^##^ *p* < 0.01, ^###^ *p* < 0.001, HSD group compared with the model group ^$^ *p* < 0.05, ^$$^ *p* < 0.01, ^$$$^ *p* < 0.001.

Comparing with control group, ELISA results showed that HK2 activity was significantly elevated in the hippocampus and cortex of mice in the model group (*p* < 0.05, *p* < 0.01), and PKM2 expression level was also significantly elevated in the model group (*p* < 0.01, *p* < 0.01). The HK2 activity in the hippocampus and cortex in the Donepezil group and HSD group was significantly reduced compared to the model group (*p* < 0.05, *p* < 0.01, *p* < 0.001, *p* < 0.01). Meanwhile, the expression level of PKM2 in the Donepezil group and HSD group was significantly reduced in the hippocampus and cortex compared to the model group (*p* < 0.01, *p* < 0.05, *p* < 0.05, *p* < 0.05) (Figures 4A–D).

### 3.5 HSD inhibits mTOR/HIF-1α signaling in the brain of SAMP8 mice

**FIGURE 5:**
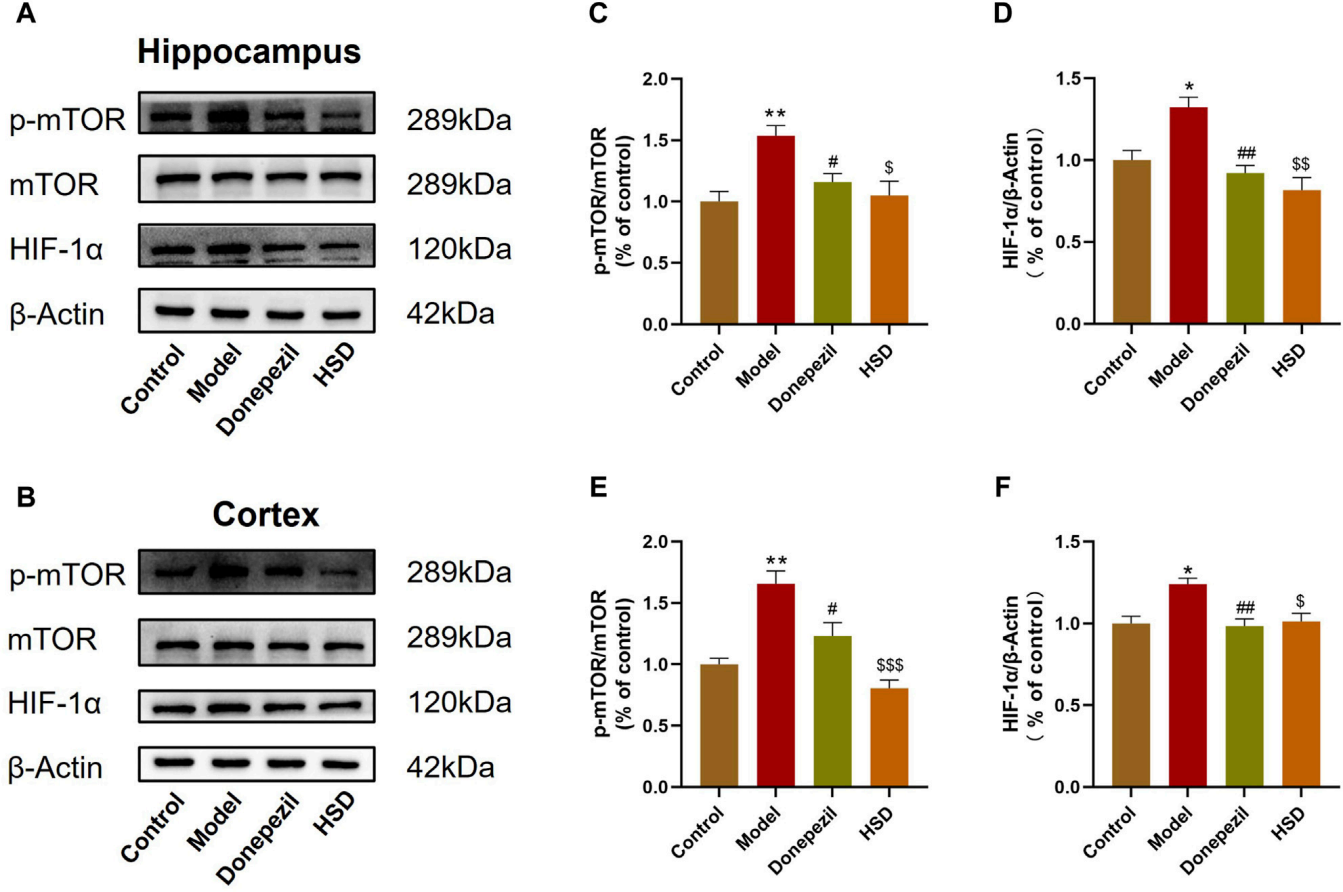
Western-blot evaluation of p-mTOR/mTOR ratio and HIF-1α protein expression levels in hippocampus and cortex of SAMP8 mice. **(A,B)** Blotting of p-mTOR, mTOR and HIF-1α protein in hippocampus and cortex of mice in each group; **(C,D)** Expression of p-mTOR/mTOR ratio and HIF-1α protein in hippocampus of mice in each group; **(E,F)** Expression of p-mTOR/mTOR ratio and HIF-1α protein in cortex of mice in each group. Model group compared with the control group ^*^ *p* < 0.05, ^**^ *p* < 0.01, ^***^ *p* < 0.001, Donepezil group compared with the model group ^#^ *p* < 0.05, ^##^ *p* < 0.01, ^###^ *p* < 0.001, HSD group compared with the model group ^$^ *p* < 0.05, ^$$^ *p* < 0.01, ^$$$^ *p* < 0.001.

The expression levels of mTOR/HIF-1α signaling pathway-related proteins in SAMP8 mice was detected. Western-blot analysis indicated that the p-mTOR/mTOR ratio in the hippocampus and cortex were significantly increased in the model group compared to the control group (*p* < 0.01, *p* < 0.01), while HIF-1α protein level was also significantly increased in the model group (*p* < 0.05, *p* < 0.05). Comparing to the model group, the p-mTOR/mTOR ratio in the hippocampus and cortex were significantly lower in the Donepezil group and HSD group (*p* < 0.05, *p* < 0.05, *p* < 0.05, *p* < 0.001), meanwhile, the expression level of HIF-1α in the hippocampus and cortex were significantly lower in the Donepezil group and HSD group (*p* < 0.01, *p* < 0.01, *p* < 0.01, *p* < 0.05) (Figures 5A–F).

### 3.6 HSD improves LPS-induced glucose metabolism reprogramming and microglial polarization by inhibiting the mTOR/HIF-1α signaling pathway in BV2 cells

**FIGURE 6:**
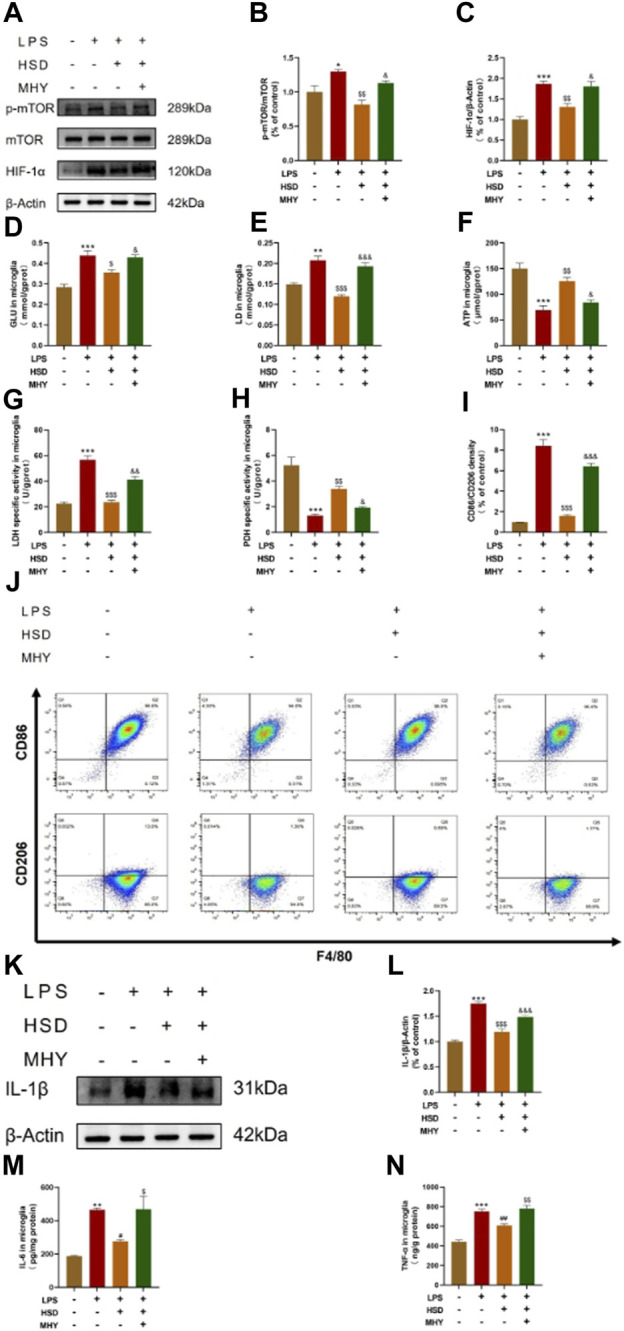
The expression levels of p-mTOR/mTOR ratio, HIF-1α, and IL-1β in BV2 cells tested by western blot, the levels of GLU, LD, ATP, LDH and PDH quantified by biochemical test kit and the levels of polarization biomarkers of CD86 and CD206 detected by flow cytometry, the expression levels of IL-6 and TNF-α in BV2 cells tested by ELISA. **(A)** Blotting of p-mTOR, mTOR and HIF-1α protein in BV2 cells in each group; **(B, C)** Expression levels of p-mTOR/mTOR ratio and HIF-1α in BV2 cells in each group. **(D)** GLU content analysis chart; **(E)** LD content analysis chart; **(F)** ATP content analysis chart; **(G)**. LDH activity analysis graph; **(H)** PDH activity analysis graph. **(I)** Flow cytometry analysis charts;**(J)** Flow cytograms of BV2 cells in each group; **(K)** Blotting of IL-1β protein in BV2 cells in each group; **(L)** Expression level of IL-1β in BV2 cells in each group. **(M, N)** Expression level of IL-6 and TNF-α in BV2 cells in each group. Model group compared with control group ^*^ *p* < 0.05, ^**^ *p* < 0.01, ^***^ *p* < 0.001, HSD-containing serum-treated group compared with the model group ^$^ *p* < 0.05, ^$$^ *p* < 0.01, ^$$$^ *p* < 0.001, Reverse validation group compared with the HSD-containing serum-treated group ^&^ *p* < 0.05, ^&&^ *p* < 0.01, ^&&&^ *p* < 0.001.

In order to investigate whether HSD-containing serum could inhibit the mTOR/HIF-1α signaling pathway in LPS-induced BV2 cells, mTOR agonist MHY was selected for reverse validation. Western-blot results showed that the p-mTOR/mTOR ratio and HIF-1α protein levels were significantly increased in the model group compared with control group (*p* < 0.05, *p* < 0.001), while which were significantly reduced in the HSD-containing serum-treated group compared with the model group (*p* < 0.01, *p* < 0.01). The p-mTOR/mTOR ratio and HIF-1α protein levels were significantly higher in the reverse validation group with MHY added compared to the HSD-containing serum-treated group (*p* < 0.05, *p* < 0.05) (Figures 6A–C).

To further observe glycolysis level and microglial polarization, LPS induced BV2 cells model were used. After the intervention of HSD-containing serum, the polarization biomarkers of BV2 cells were evaluated by glucose metabolites were detected by GLU, LD, ATP, LDH and PDH test kits and flow cytometry. Figures 6D–H demonstrated that the levels of GLU and LD, and the activity of LDH were significantly elevated in the model group compared to the control group (*p* < 0.001, *p* < 0.01, *p* < 0.001), while the expression level of ATP and the activity of PDH were significantly decreased (*p* < 0.001, *p* < 0.001). The levels of GLU and LD, as well as the activity of LDH, were found to be significantly decreased in the HSD-containing serum-treated group compared to the model group (*p* < 0.05, *p* < 0.001, *p* < 0.001), while the level of ATP and the activity of PDH were significantly increased (*p* < 0.01, *p* < 0.01). Interestingly, the levels of GLU and LD and the activity of LDH were found to be markedly elevated in the reverse validation group compared to the HSD-containing serum-treated group (*p* < 0.05, *p* < 0.001, *p* < 0.01), while the level of ATP and the activity of PDH were significantly decreased in the reverse validation group (*p* < 0.05, *p* < 0.05). The results showed that the ratio of CD86 to CD206 of BV2 cells was significantly increased in the model group compared to the control group. (*p* < 0.001). The CD86/CD206 ratio was significantly decreased in the HSD-containing serum-treated group compared to the model group (*p* < 0.001), while which was significantly increased in the reverse validation group compared to the HSD-containing serum-treated group (*p* < 0.001) (Figures 6I, J). Furthermore, western-blot results showed that the expression level of IL-1β was significantly increased in the model group compared with control group (*p* < 0.001), while which were substantially decreased in the HSD-containing serum-treated group in comparison with the model group (*p* < 0.001). The protein expression level of IL-1β was significantly higher in the reverse validation group with MHY added compared to the HSD-containing serum-treated group (*p* < 0.001) (Figures 6K, L). ELISA results showed that the expression levels of IL-6 and TNF-α were significantly increased in the model group compared with control group (*p* < 0.01, *p* < 0.001), while which were substantially decreased in the HSD-containing serum-treated group in comparison with the model group (*p* < 0.05, *p* < 0.01). The protein expression levels of IL-1β and TNF-α were significantly higher in the reverse validation group with MHY added group compared to the HSD-containing serum-treated group (*p* < 0.05, *p* < 0.01) (Figures 6M, N).

## 4 Discussion

HSD is a Chinese herbal compound which has been reported to be clinically effective in the treatment of AD (Zhai et al., 2022). The present study demonstrated that HSD improved learning and memory ability and alleviated cognitive dysfunction in SAMP8 mice. The mechanism by which HSD alleviated AD symptoms may be achieved by inhibiting of the mTOR/HIF-1α signaling pathway, modulating BV2 glycolytic reprogramming, and attenuating neuroinflammation.

SAMP8 is an age-related AD mouse model with kidney essence deficiency (Song et al., 2022). In order to observe the effects of HSD on the learning and memory abilities and cognitive functions of SAMP8 mice, we first conducted behavioral experiments. The results of behavioral studies demonstrated that SAMP8 mice exhibited decreased learning and memory abilities and cognitive dysfunction. Interestingly, the learning and memory abilities and spatial exploration abilities of the SAMP8 mice were improved after the intervention of HSD. Aβ deposition is a classical pathologic indicator of AD (Li et al., 2020). IHC results indicate that Aβ plaque deposition is present in the brains of SAMP8 mice, and HSD reduced Aβ deposition and alleviated AD pathology.

Neuroinflammation plays a significant role in the pathogenesis of AD (Muhammad et al., 2019). Altered glucose metabolism in microglia influences neuroinflammation, in which, the glucose metabolism of microglial cells will reprogram from OXPHOS to aerobic glycolysis, with HK2 and PKM2 acting as rate-limiting enzymes in glycolysis. This study showed that HSD can reduce the activity of glycolytic rate-limiting enzyme HK2 and the expression level of PKM2 in the hippocampus and cortex of SAMP8 mice, decrease the expression levels of M1 markers of CD86 and pro-inflammatory factor IL-1β, IL-6 and TNF-α and increasing the expression levels of M2 markers of Arg-1, suggesting that HSD weakened the aerobic glycolysis and exert anti-inflammatory functions. Glycolysis mediated by the mTOR/HIF-1α signaling pathway enhances inflammatory responses (Cheng et al., 2014). In the vitro study, LPS -induced microglial activation is commonly used in AD (Cheng et al., 2021; Wang et al., 2022). To better verify the role of HSD in microglial in AD, LPS induced BV2 cells were conducted to reverse validation along with the mTOR activator MHY. LPS binds to toll-like receptors (TLRs) on the surface of microglial, leading to elevated level of glycolysis and contributing to a pro-inflammatory response (Orihuela et al., 2016). The results of this study showed that the ratio of CD86 to CD206 and the level of IL-1β, IL-6 and TNF-α expression increased in LPS-induced BV2 cells, which enhanced the inflammatory response. HSD-containing serum reduced the ratio of CD86 to CD206 and the level of IL-1β, IL-6 and TNF-α expression and attenuated the inflammatory response, while MHY reversed this effect. LPS-induced BV2 cells utilize glycolysis for glucose metabolism in response to pro-inflammatory factors (Devanney et al., 2020), which increases GLU uptake and LD production (Orihuela et al., 2016), and decreases ATP level (Zhang et al., 2021). LDH is a key enzyme in aerobic glycolysis, catalysing the conversion of pyruvate to LD (Sharma et al., 2022), while PDH is a key branching enzyme in glucose metabolism that enhances the level of oxidative phosphorylation (Kono et al., 2018). Our results indicate that HSD-containing serum can reduce GLU and LD levels, as well as LDH activity, and increase ATP level and PDH activity in LPS-induced BV2 cells. It is suggested that HSD can decrease glycolysis level in microglia, increase oxidative phosphorylation level, and these effects are reversed by MHY.

**FIGURE 7:**
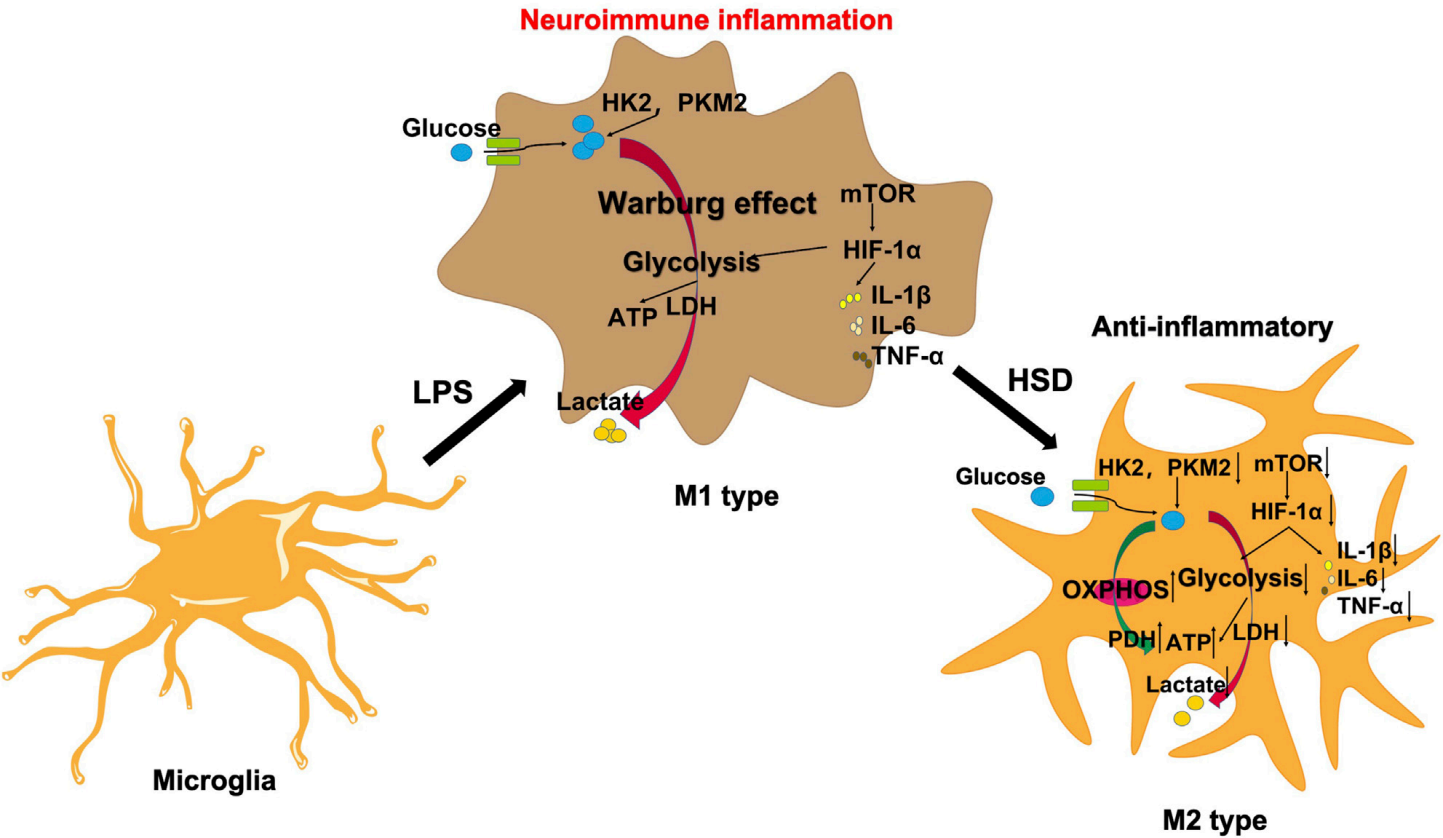
HSD regulates microglial glucose metabolism reprogramming to alleviate AD neuroinflammation through the mTOR/HIF-1α signaling pathway.

The mTOR/HIF-1α signaling pathway plays a crucial role in the metabolic reprogramming of microglia (Guan et al., 2023). In this study, we observed that HSD-containing serum can reduce the levels of p-mTOR/mTOR ratio and HIF-1α proteins which was reversed by MHY. Therefore, HSD can regulate the glycolytic reprogramming of microglial and alleviate neuroinflammation by inhibiting the mTOR/HIF-1α signaling pathway (Figure 7).

## 5 Conclusion

This study indicate that HSD can regulate glucose metabolism reprogramming in microglia through the mTOR/HIF-1α signaling pathway, alleviate neuroinflammation, reduce Aβ pathology, and improve learning and memory deficits in AD mice. These findings offer a scientific support for the potential use of HSD in clinical applications. Furthermore, further identification of the anti-AD active components in HSD needs to be investigated to provide scientific evidence and theoretical guidance for the development of novel anti-AD drugs.

## Data Availability

The raw data supporting the conclusions of this article will be made available by the authors, without undue reservation.

## Abbreviations

AD, Alzheimer’s Disease; HSD, Huanshaodan; Aβ, amyloid-beta; ELISA, Enzyme-Linked Immunosorbent Assay; HK2, Hexokinase 2; PKM2, Pyruvate kinase M2; LPS, Lipopolysaccharide; GLU, glucose; LD, lactic acid; LDH, lactate dehydrogenase; PDH, pyruvate dehydrogenase; OXPHOS, oxidative phosphorylation; bFGF, fibroblast growth factor; NGF, nerve growth factor; MHY, MHY1485.
